# Probabilistic risk assessment of solar particle events considering the cost of countermeasures to reduce the aviation radiation dose

**DOI:** 10.1038/s41598-021-95235-9

**Published:** 2021-09-02

**Authors:** Moe Fujita, Tatsuhiko Sato, Susumu Saito, Yosuke Yamashiki

**Affiliations:** 1 Data Solution Group, Aioi Nissay Dowa Insurance Co., Ltd, Shibuya-ku, Tokyo Japan; 2 Nuclear Science and Engineering Center, Japan Atomic Energy Agency, Tokai, Japan; 3 Electronic Navigation Research Institute, National Institute of Maritime, Port and Aviation Technology, Tokyo, Japan; 4 SIC Human Spaceology Center, Graduate School of Advanced Integrated Studies in Human Survivability, Kyoto University, Kyoto, Japan

**Keywords:** Solar physics, Risk factors

## Abstract

Cosmic-ray exposure to flight crews and passengers, which is called aviation radiation exposure, is an important topic in radiological protection, particularly for solar energetic particles (SEP). We therefore assessed the risks associated with the countermeasure costs to reduce SEP doses and dose rates for eight flight routes during five ground level enhancements (GLE). A four-dimensional dose-rate database developed by the Warning System for Aviation Exposure to Solar Energetic Particles, WASAVIES, was employed in the SEP dose evaluation. As for the cost estimation, we considered two countermeasures; one is the cancellation of the flight, and the other is the reduction of flight altitudes. Then, we estimated the annual occurrence frequency of significant GLE events that would bring the maximum flight route dose and dose rate over 1.0 mSv and 80 μSv/h, respectively, based on past records of GLE as well as historically large events observed by the cosmogenic nuclide concentrations in tree rings and ice cores. Our calculations suggest that GLE events of a magnitude sufficient to exceed the above dose and dose rate thresholds, requiring a change in flight conditions, occur once every 47 and 17 years, respectively, and their conservatively-estimated annual risks associated with the countermeasure costs are up to around 1.5 thousand USD in the cases of daily-operated long-distance flights.

## Introduction

Cosmic-ray exposure is an important topic for aviation workers, such as cabin attendants and pilots in most flight companies. In principle, the higher the altitude and latitude of the plane, the higher the dose rate of radiation when flying. Therefore, the International Committee on Radiological Protection (ICRP) recognizes the cosmic-ray exposure to aircrews as an occupational hazard^1^. In addition, ICRP provided an updated guidance on radiological protection from aircrew exposure, considering the current ICRP system of radiological protection^2^. In response to these publications, the International Civil Aviation Organization (ICAO) has recently decided to use radiation dose as mandatory information requested from space weather information providers^3^.

Two primary cosmic-ray sources can contribute to aviation radiation exposure: galactic cosmic rays (GCR) and solar energetic particles (SEP). The GCR fluxes are relatively stable and predictable compared to of SEP, and their dose rates are always low—below 10 μSv/h at the conventional flight altitude of 12 km^4^. By contrast, SEP fluxes suddenly increase when a large solar particle event (SPE) occurs, and their dose rates occasionally become very high—more than 2 mSv/h^5^, though the duration of such high dose rate events are generally short. Considering that ICRP recommends suppressing the dose to an embryo/fetus below that of about 1 mSv^2^, it is desirable to take adequate actions such as reduction of the flight altitude during such large SPEs.

There are two major methods for detecting a SPE: one is based on high-energy proton detectors mounted on Geostationary Operational Environmental Satellites (GOES), and the other is based on neutron monitors on the Earth’s surface. The former can detect SEPs directly by measuring proton fluxes above 1 MeV, while the latter detects SEPs indirectly by measuring secondary neutrons generated through nuclear interactions induced by SEPs in the atmosphere. SPEs with a significant increase in neutron monitor count rates are rarely observed in comparison to those with an increase in the GOES proton fluxes, because most SPEs do not emit high-energy protons (*E* > 450 MeV) that can create neutrons reaching the Earth’s surface. These events are called ground-level enhancement (GLE), and only 72 of them have been recorded over eight decades of observation. Using the GOES and/or neutron monitor data, several systems have been developed to issue an alert to SEP exposure or provide the information on SEP doses at flight altitudes^6–11^.

If an airline company takes actions to reduce aviation doses in response to an alert issued by these systems, it is necessary to estimate its costs. A potential mitigation procedure is a reduction of flight altitude, and Matthiä et al. discussed its economic impact^12^. However, the discussion was based on calculated aviation dose for a certain flight condition, which was a transatlantic flight on December 13th, 2006 from Seattle to Cologne, during which GLE 70 occurred. Yamashiki et al.^13^, also made a cost estimation of aviation radiation exposure for a short flight distance (US domestic flight) using X-ray flux (W/m^2^) based on GOES satellite measurements as the index of the magnitude of an SPE, but the spatial variation of the SEP dose rates were not considered in their estimation. In order to generalize the cost and develop insurance for aviation radiation exposure, estimations of aviation doses for various flight conditions are indispensable. The frequency of the occurrence of SPEs that require a mitigation procedure must also be evaluated.

With these situations in mind, we calculated the maximum doses and dose rates due to SEP exposure for eight flight routes with two cruise altitudes during five GLE events, by integrating the four-dimensional aviation doses calculated by WASAVIES^9,10^ Based on the results, the annual occurrence frequency that the total doses exceed 1 mSv or the dose rates exceed 80 μSv/h were estimated by scaling the magnitude of the GLE using the event-integrated intensity (EII) proposed by Asvestari^14^ or peak-event intensity (PEI) proposed in this study. Note that 80 μSv/h is the threshold dose rate that is classified as “severe” exposure in the Space Weather D-index^15,16^ and the ICAO space weather advisory information^3^. Then, the cancellation and extra fuel costs were estimated in order to help to design an insurance system for airline companies to protect against elevated aviation radiation dose.

## Materials and method

### Estimation of flight route doses during GLE events

Four-dimensional (Three spatial dimensions and one temporal dimension) dose rate data for 5 different GLE events (GLE60, 69–72) were prepared using WASAVIES: WArning System for AVIation Exposure to Solar energetic particles. This can determine the aviation dose during a GLE anywhere in the atmosphere in 5-min intervals using databases based on SEP transport simulations from the Sun to the ground level of the Earth. In WASAVIES, it is assumed that the SEP fluence and its temporal variation generated around the Sun can be simply expressed by a power function of energy and the inverse Gaussian distribution of time, respectively. Then, the database of the SEP fluences at 1 astronomical unit (AU) was prepared by solving the one-dimensional focused transport equation for 6 power indexes and 3 shape parameters^17^. In addition to the power index and shape parameter, the total fluence and tilt angle of the SEP incident to the Earth must be determined in order to characterize GLE, and their numerical values are evaluated in real time using the GOES proton fluxes and the count rates of several neutron monitors on the ground. Note that the evaluated parameters vary with time, and thus, the temporal variations of the GLE characteristics such as hard and soft spectra of SEP at the increasing and decreasing phases of GLE, respectively, can be considered in WASAVIES. For spatial resolution, the atmosphere was divided into 28 altitude layers, and the data for each altitude was an average value at intervals of 15 degrees for longitude and 10 degrees for latitude. The intricated latitude, longitude, and altitude dependences of the dose rates were reproduced by developing the databases of SEP trajectories in the magnetosphere using the empirical geomagnetic field model T89^18^ and the airshower simulation performed by the PHITS code^19^. The aviation doses due to GCR exposure can also be calculated in the system, using the PARMA model^4^.

Eight long-distance and high-latitude flight routes were selected in this study for investigating aviation doses, and they are summarized in Table 1. The information on each flight route and time are taken from the Japanese Internet System for Calculation of Aviation Route Doses, JISCARD^20^, which assumes that the aircraft flies on the great circle routes at a constant cruise altitude (9 km or 12 km). The average speeds for each route are between 531 miles/h (855 km/h) and 467 miles/h (752 km/h), which was calculated considering the drag force during ascent and descent as well as cruising with a stable speed at the altitude of 12 km. For the alternative cruise altitude of 9 km, we simply applied the same flight path and speed with 12 km in order to unify and simplify the discussion along the flare time. This should be improved in further studies. Then, we calculated the dose rates on each flight path at 5-min intervals based on the four-dimensional (latitude, longitude, altitude, and time) dose rate data evaluated by WASAVIES. The total doses obtained for each flight were estimated by simply integrating the calculated dose rates with respect to the entire flight duration. In this estimation, we made a series of hypothetical timelines for the departure of each flight, setting before, during, and after the onset of each GLE event in order to find the flight schedule to give the maximum total dose.

**Table 1:** Information on 8 selected flight routes and their fuel and cancellation costs.

Flight ID	Departure	Arrival	Distance		Time (h)	Cost (1000 USD)			
			(Mile)	(km)		Fuel@12 km	Fuel@9 km	Fuel increase (@9 km–@12 km)	Cancellation
LAX_LHR	Los Angeles	London	5488	8832	10.6	51	63	12	97
SYD_EZE	Sydney	Buenos Aires	7368	11,858	13.9	68	84	16	67
SFO_LHR	San Francisco	London	5399	8689	10.4	50	62	12	98
NRT_LHR	Tokyo	London	6009	9671	12.9	62	77	15	78
SYD_GIG	Sydney	Rio de Janeiro	8463	13,620	15.9	77	95	18	52
SYD_LIM	Sydney	Lima	8006	12,884	15.1	73	90	17	59
SYD_CPT	Sydney	Cape Town	6882	11,075	14.6	71	87	16	62
NRT_JFK	Tokyo	New York	6784	10,918	12.9	63	77	13	76

The fuel costs were estimated for a B747-400 with weight of 500,000 lbs (226,796 kg) at the altitudes of 9 and 12 km with the respective fuel consumption rate of 21,000 lbs (9525 kg)/h and 17,000 lbs (7711 kg)/h, respectively found in literature^21^. They are shown in Table 1. Note that the price of fuel was set to 0.284 USD/lb (0.626 USD/kg) in this estimation. Cancellation costs were estimated based on the methodology and cost parameter introduced by Marks^22^ based on statistics of airlines in the USA. The cancellation costs consist of three major components. One is the base incremental cost which includes costs for crew, maintenance, and airport related costs. Second is the net offset cost which become unnecessary, such as fuel that would have been burnt, landing fees, and overflight fees. These are negative costs. Third is the commercial cost which is caused by ticket refunds and displacement of revenue associated with rebooking of passengers. Accommodation cost for passengers were not considered, because the SPE can be considered as an uncontrollable event for which airlines are not responsible. The total cancellation costs are the sum of the cancellation costs of the particular flight and its subsequent flight which will also be cancelled. We used the parameters shown by Marks^22^ for a long-haul international flight with two-cabin-class configuration. The ticket prices are assumed to be on average the same for all the routes. The fuel related parameters were replaced by those used for the fuel costs calculation at 9 and 12 km. The results are shown in Table 1. It should be noted that the cost may be different for airlines operating in different regions.

**Figure 1:**
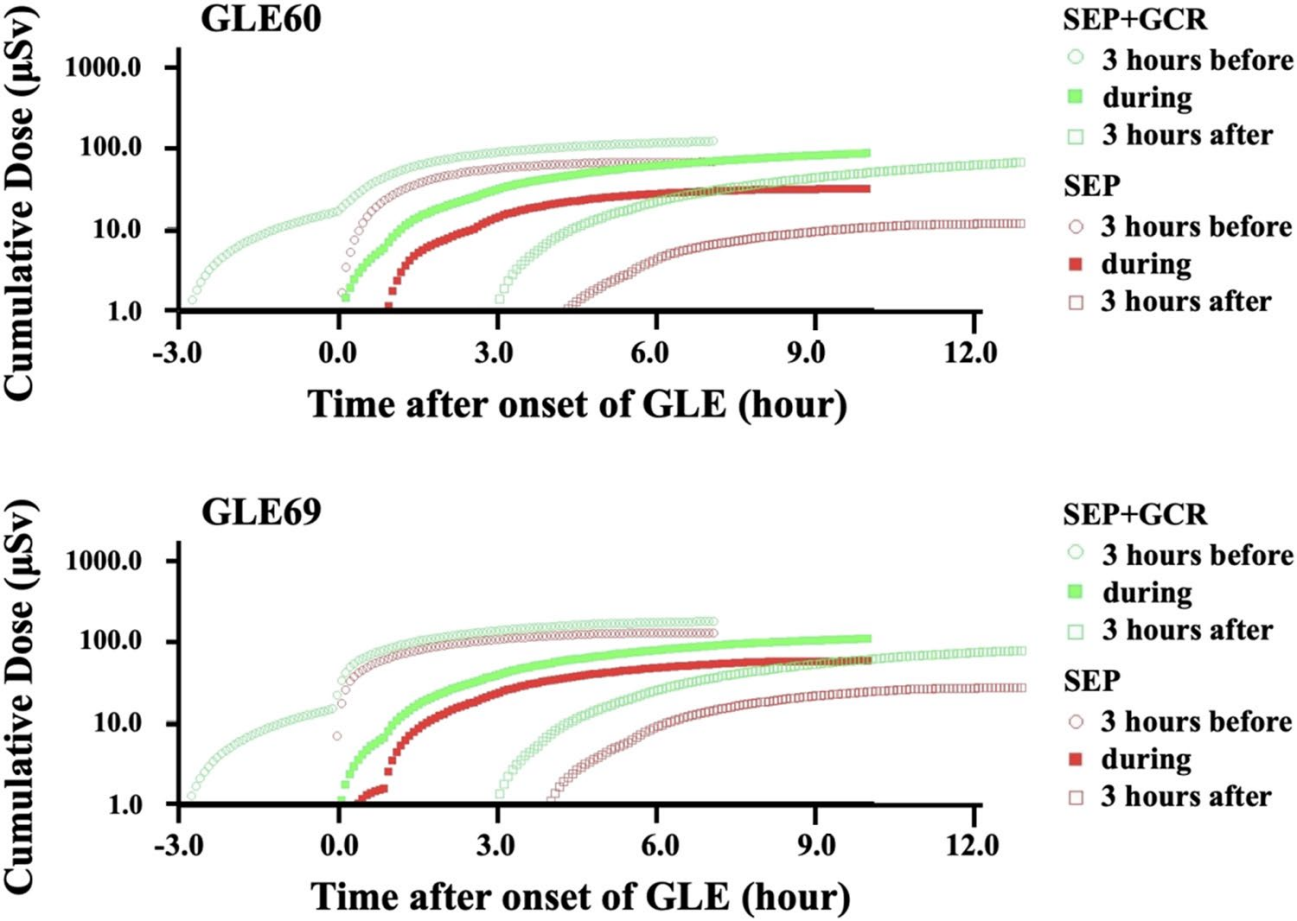
Calculated cumulative dose for flights from Los Angeles to London departing 3 h before, during, and 3 h after the onset of GLE 60 and 69, respectively. The cruise altitude was set to 12 km in this calculation.

Figure 1 shows the temporal changes in cumulative dose for the flight route of Los Angeles to London (LAX-LHR) departing 3 h before, during, and 3 h after the onset of GLE 60 and 69, respectively. For the numerical simulation, we evaluated in 5 min sequences for the duration of 24 h. Note that all doses calculated in this study are the effective dose based on the definition of the 2007 recommendations of ICRP^23^. The green and red dots represent the cumulative dose in μSv for a single flight path with different departure times, including and excluding the GCR dose component, respectively. In this case, a flight departing approximately 3 h before the onset of GLE gives the maximum dose for both GLEs. This calculation has been accomplished for all flight routes, with different GLEs under different flight altitudes.

### Calculation of the annual frequency of GLE

For representing GLE magnitude, Asvestari et al.^14^ proposed using EII, which is defined as the integral of the excess above the GCR background over the entire duration of the event. It corresponds to the total fluence of SEPs with energy sufficient to cause an atmospheric cascade (several hundred MeV). The EII in the unit of %*h for 48 GLEs in the past 70 years were evaluated by Asvestari et al.^14^, using GLE records of polar sea-level neutron monitors. Although the count rates of the neutron monitors located only at the polar region were used in the determination of EII, it can be used as an index for representing the global increase of aviation dose level for the entire GLE period because the SEP doses hardly increase at lower latitude regions.

On the other hand, the index for representing the highest SEP dose rate at a certain location in the world can also be useful in considering insurance for aviation radiation exposure. We therefore introduced PEI, which is the highest count rate (counts per minute) increase above GCR background count rate seen among all neutron monitors except for the South Pole station. The reason for excluding the data from the South Pole is that the altitude and latitude of the station are so high that the count rates cannot be directly compared with the data for other stations. The numerical values of PEI for each GLE were obtained from the count rates of all available neutron monitors provided on the Oulu station website (http://cosmicrays.oulu.fi/).

**Figure 2:**
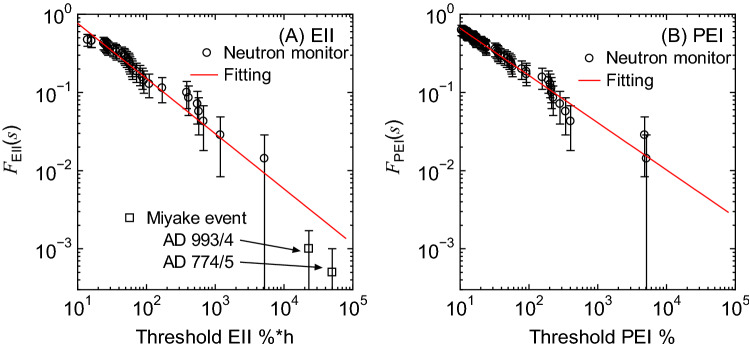
Annual frequency of the occurrence of GLE with (**A**) EII or (**B**) PEI above certain threshold value *s*. The error bars were simply estimated from ${F}_{\rm{EII}}(s)/\sqrt{{N}_{\rm{GLE}}}$ and ${F}_{\rm{PEI}}(s)/\sqrt{{N}_{\rm{GLE}}}$, where *N*_GLE_ is the overall number of GLE contributing to the data.

Then, we calculated the annual frequencies of the occurrence of GLE with EII or PEI above a certain threshold value *s*, *F*_EII_(*s*) or *F*_PEI_(*s*), by counting the number of corresponding GLEs divided by 70. For example, 10 GLEs with EII above 92% × h were observed in the past 70 years, *F*_EII_(92) can be determined to be 0.143, i.e. 10/70. The calculated frequencies are shown in Fig. 2 as a function of the threshold EII or PEI. In the plots, we excluded GLEs with EII or PEI values less than 10%*h or 10% because SPEs with such low EII or PEI might not be detected as a GLE.

In addition, recent investigations on the cosmogenic nuclide concentrations in tree rings and ice cores have revealed that extremely large SPEs with hard SEP spectra occurred in AD 774/5 and AD 993/4^24,25^. The total SEP fluences during these events were estimated to be 119–141 and 51–68 times higher than those during GLE 69^26^. We therefore assumed that the SPE having EII 59.5 and 130 times higher than that of GLE 69, 385%*h, could occur twice and once per 2000 years respectively, and added the frequencies of *F*_EII_(22,908) = 0.001 and *F*_EII_(50,050) = 0.0005 in Fig. 2. On the other hand, PEI of those historical events has not been investigated, and they are not included in Fig. 2. It should be mentioned that those historical events might consist of multiple SPEs. However, this fact does not result in the large uncertainties of the fitting described below because the magnitude and frequency of each SPE become smaller and higher, respectively. For example, if 10 SPEs with an equal magnitude would have occurred during those historical events, the corresponding frequencies would be *F*_EII_(2290.8) = 0.01 and *F*_EII_(5005) = 0.005, which are still consistent with the fitting results shown in Fig. 2.

In this study, *F*_EII_(*s*_EII_) is assumed to follow the power-law function of *s*_EII_, as written by:

1 $$ F_{{{\rm{EII}}}} \left( {s_{{{\rm{EII}}}} } \right) = 10^{{\left\{ {A_{{{\rm{EII}}}} \log _{{10}} \left({s_{{{\rm{EII}}}} } \right)+ B_{{{\rm{EII}}}} } \right\}}}, $$

where *A*_EII_ and *B*_EII_ are the fitting parameters obtained from the scatter plot shown in Fig. 2 and their numerical values are − 0.706 and 0.591, respectively. In a similar manner, *F*_PEI_(*s*_PEI_) can be calculated by

2 $$F_{{{\text{PEI}}}} (s_{{{\text{PEI}}}} ) = 10^{{\{ A_{{{\text{PEI}}}} {\text{log}}_{{10}} \left( {s_{{{\text{PEI}}}} } \right) + B_{{{\text{PEI}}}} \} }} . $$

The numerical values of *A*_PEI_ and *B*_PEI_ are evaluated to be − 0.604 and 0.425, respectively.

Then, the annual frequency that the maximum flight route dose exceeds the threshold dose, *D*_thre_, for a certain flight route *i* estimated from the *j*th GLE event can be calculated from Eq. (1) by substituting ${s}_{\rm{EII},j}{D}_{\rm{thre}}/{D}_{i,j}$ into *s*_EII_, i.e. ${F}_{\rm{EII}}\left({s}_{\rm{EII},j}{D}_{\rm{thre}}/{D}_{i,j}\right)$, where *s*_EII,*j*_ is the EII value for the *j*th GLE event and *D*_*i*,*j*_ is the maximum SEP dose for a flight route *i* during the *j*th GLE event. For example, if the maximum SEP dose for a certain flight route *i* during the *j*th GLE event with EII = 1000%*h is 0.1 mSv, *F*_EII_ for *D*_thre_ = 1.0 mSv is estimated to be 10^(−0.706×log10(1000(1.0/0.1))+0.591)^ = 0.00584. In a similar manner, the annual frequency that the maximum flight route dose rate exceeds the threshold dose rate, ${\dot{D}}_{\rm{thre}}$ can be calculated from Eq. (2) by substituting ${s}_{\rm{PEI},j}{\dot{D}}_{\rm{thre}}/{\dot{D}}_{i,j}$ into *s*_PEI_, i.e. ${F}_{\rm{PEI}}\left({s}_{\rm{PEI},j}{\dot{D}}_{\rm{thre}}/{\dot{D}}_{i,j}\right)$, where ${\dot{D}}_{i,j}$ is the maximum SEP dose rate for flight route *i* during the *j*th GLE event. Note that these threshold dose and dose rate do not include the contribution from GCR.

### Probabilistic risk estimation

In the risk estimation associated with aviation exposure due to SEPs, we assumed two simple and fundamental solutions as countermeasures to reduce the SEP dose; one is the cancellation of the flight when the maximum dose or dose rate exceeds *D*_thre_ or ${\dot{D}}_{\rm{thre}}$ at the flight altitude of 9 km, and the other is to lower the cruise altitudes from 12 to 9 km when the maximum dose or dose rate exceeds the threshold level at 12 km but does not at 9 km. Here, we simply assume that cruising at altitudes lower than 9 km would not be practical, because the aircraft may suffer from turbulence induced by upwelling flow more frequently and from increased air drag due to exponentially increasing air density than flying at a higher altitude. A more practical solution should be addressed in a future study, together with the potential countermeasures for en route flights, such as routing to lower latitudes, temporarily lowering flight altitude, and emergency landing, which should be assessed with a more realistic aircraft performance model and considerations from the viewpoint of air traffic management^27,28^.

Based on this strategy, the annual risk associated with avoiding the maximum dose of *D*_thre_ for flight routes *i* estimated from the *j*th GLE event, *R*_*i*,*j*_(*D*_thre_), was calculated as follows:

3 $$ \begin{aligned}{R}_{i,j}\left({D}_{\rm{thre}}\right)&=\left[{F}_{\rm{EII}}\left({s}_{\rm{EII},j}{D}_{\rm{thre}}/{D}_{i@12\rm{km},j}\right)-{F}_{\rm{EII}}\left({s}_{\rm{EII},j}{D}_{\rm{thre}}/{D}_{i@9\rm{km},j}\right)\right]\left({C}_{i@9\rm{km}}-{C}_{i@12\rm{km}}\right)\\&\quad+{F}_{\rm{EII}}\left({s}_{\rm{EII},j}{D}_{\rm{thre}}/{D}_{i@9\rm{km},j}\right){C}_{i\rm{Cancel}},\end{aligned} $$

where ${D}_{i@9\rm{km},j}$ and ${D}_{i@12\rm{km},j}$ are the maximum SEP doses for flight route *i* at the cruise altitudes of 9 km and 12 km, respectively, *C*_*i*@9 km_ and *C*_*i*@12 km_ are the fuel costs of flight route *i* at the cruise altitudes of 9 km and 12 km, respectively, and *C*_*i*Cancel_ is the cancellation cost given in Table 1. The first term represents the risk associated with the extra fuel cost when a flight can be operated at an altitude of 9 km but not at 12 km, and the second term represents the risk associated with the cancellation cost when a flight cannot be operated even at an altitude of 9 km. In the same manner, the annual risk associated with avoiding the maximum dose rate of ${\dot{D}}_{\rm{thre}}$ for flight routes *i* estimated from the *j*th GLE event, ${R}_{i,j}\left({\dot{D}}_{\rm{thre}}\right)$, was calculated as follows:

4 $$ \begin{aligned}{R}_{i,j}\left({\dot{D}}_{\rm{thre}}\right)&=\left[{F}_{\rm{PEI}}\left({s}_{\rm{PEI},j}{\dot{D}}_{\rm{thre}}/{\dot{D}}_{i@12\rm{km},j}\right)-{F}_{\rm{PEI}}\left({s}_{\rm{PEI},j}{\dot{D}}_{\rm{thre}}/{\dot{D}}_{i@9\rm{km},j}\right)\right]\left({C}_{i@9\rm{km}}-{C}_{i@12\rm{km}}\right)\\&\quad+{F}_{\rm{PEI}}\left({s}_{\rm{PEI},j}{\dot{D}}_{\rm{thre}}/{\dot{D}}_{i@9\rm{km},j}\right){C}_{i\rm{Cancel},}\end{aligned} $$

where ${\dot{D}}_{i@9\rm{km},j}$ and ${\dot{D}}_{i@12\rm{km},j}$ are the maximum SEP dose rates for flight route *i* at the cruise altitudes of 9 km and 12 km, respectively. We calculated the risk for *D*_thre_ = 1 mSv and ${\dot{D}}_{\rm{thre}}$ = 80 μSv/h, which are the annual dose limitation of public exposure in planned exposure situations recommended by ICRP and the threshold dose rate that is classified as “severe” exposure in the Space Weather D-index, respectively.

It should be noted that Eqs. (3) and (4) give conservative estimates of the risks based on the maximum dose or dose rates for each flight route, i.e., they intrinsically assume that the worst-case-scenario flight is always scheduled when a GLE occurs. To reduce the conservativeness, we introduced the scaling factor for considering the probability of scheduling the worst (or equivalent) scenario flight, which should complicatedly depend on the flight route and frequency, as well as the temporal and spatial variations of the SEP dose rates. For simplicity, we presume that the worst scenario always occurs when the GLE onset is during the cruise time of a flight in this study, and approximate the scaling factor by the product of the number of annual scheduled flights per route and cruise time per flight divided by the total time per year. For example, the scaling factor is determined from the cruise time in hours divided by 24 in the case of daily-operated flight. Further analysis for more precisely evaluating the scaling factor must be performed in the future.

## Results and discussion

Table 2 shows the maximum doses and dose rates due to SEP exposure for eight selected flight routes at a 12 km altitude during five GLE events, ${D}_{i@12\rm{km},j}$ and ${\dot{D}}_{i@12\rm{km},j}$, respectively, estimated from the four-dimensional dose rate data, which are characterized by the spectral power index, temporal shape parameter, total fluence, and tilt angle of SEP incident to the Earth evaluated by WASAVIES. In general, the SEP dose and dose rates during GLE 69 are the largest among the five selected GLE events, particularly for the dose rate. This is because GLE 69 was one of the largest and the most impulsive events that have occurred since they have been reliably recorded by neutron monitors.

**Table 2:** Maximum doses (μSv) and dose rates (μSv/h) due to SEP exposure for eight selected flight routes at 12 km and 9 km altitude during five GLE events estimated from the four-dimensional dose rate data calculated by WASAVIES.

Flight ID	LAX_LHR	SYD_EZE	SFO_LHR	NRT_LHR	SYD_GIG	SYD_LIM	SYD_CPT	JFK_NRT
GLE60	EII = 170 (%*h), PEI = 149%, *Duration = 34 h							
Dose @ 12 km (μSv)	67.7	42.2	68.3	48.6	56.1	12.5	43.2	45.6
Dose @ 9 km (μSv)	25.5	16.1	25.6	18.4	21.1	4.81	16.4	17.6
Dose rate @ 12 km (μSv/h)	36.7	21.2	36.7	33.1	31.7	8.22	24.7	27.6
Dose rate @ 9 km (μSv/h)	14.2	7.91	14.2	12.7	11.9	3.12	9.34	10.9

GLE69	EII = 385 (%*h), PEI = 2650%, *Duration = 36 h							
Dose @ 12 km (μSv)	117	96.3	104	121	125	41.5	124	120
Dose @ 9 km (μSv)	41.9	35.4	37.6	44.6	45.8	16.0	45.3	43.9
Dose Rate @ 12 km (μSv/h)	200	241	200	273	241	54.6	127	148
Dose rate @ 9 km (μSv/h)	73.5	91.4	73.5	101	91.4	20.0	49.8	54.1

GLE70	EII = 62 (%*h), PEI = 92%, *Duration = 31 h							
Dose @ 12 km (μSv)	21.2	33.6	21.8	31.1	35.3	10.6	39.0	32.6
Dose @ 9 km (μSv)	7.64	12.8	7.86	11.9	13.0	4.58	14.6	11.9
Dose rate @ 12 km (μSv/h)	8.40	26.1	7.51	18.1	26.1	9.65	27.2	21.1
Dose rate @ 9 km (μSv/h)	3.29	10.8	3.10	7.79	10.8	4.43	11.2	8.46

GLE71	EII = 10 (%*h), PEI = 16%, *Duration = 14 h							
Dose @ 12 km (μSv)	3.75	5.77	4.41	5.00	5.63	2.04	6.76	5.74
Dose @ 9 km (μSv)	1.34	2.11	1.56	1.85	2.07	0.84	2.46	2.10
Dose rate @ 12 km (μSv/h)	1.90	5.03	3.67	4.24	5.03	2.08	5.03	4.78
Dose rate @ 9 km (μSv/h)	0.78	1.90	1.38	1.62	1.90	0.87	1.90	1.81

GLE72	EII = 9.5 (%*h), PEI = 16%, *Duration = 54 h							
Dose @ 12 km (μSv)	10.9	9.60	12.1	10.7	13.0	1.54	12.9	12.5
Dose @ 9 km (μSv)	3.62	3.17	3.96	3.28	4.12	0.57	3.98	3.92
Dose rate @ 12 km (μSv/h)	3.27	2.80	3.43	2.42	2.94	1.06	2.40	3.46
Dose rate @ 9 km (μSv/h)	1.18	0.96	1.20	0.65	1.01	0.39	0.69	1.20

*Approximate duration with GOES proton (E > 100 MeV) flux over 1 (/cm^2^/sr/s).

Table 3 shows the annual occurrence frequencies of the maximum SEP dose exceeding 1 mSv at the cruise altitudes of 12 km and 9 km, i.e. ${F}_{\rm{EII}}\left({s}_{\rm{EII},j}{D}_{\rm{thre}}/{D}_{i@12\rm{km},j}\right)$ and ${F}_{\rm{EII}}\left({s}_{\rm{EII},j}{D}_{\rm{thre}}/{D}_{i@9\rm{km},j}\right)$ for *D*_thre_ = 1 mSv, estimated from the calculated SEP doses given in Table 2 in combination with the regression line obtained from EII as shown in Fig. 1. For example, the calculated maximum SEP dose for LAX_LHR at 12 km during GLE 60 (EII = 170%*h) is 0.0677 mSv, indicating that GLE with EII = 170/0.0677 = 2511%*h can give the SEP dose of 1 mSv for the flight route. Then, the frequency of the occurrence of a GLE event with EII above 2511%*h is estimated to be 10^(−0.706*log10(2511)+0.591)^ = 0.0155 per year. The annual occurrence frequencies of the maximum SEP dose rate exceeding 80 μSv/h at the cruise altitudes of 12 and 9 km, i.e. ${F}_{\rm{PEI}}\left({s}_{\rm{PEI},j}{\dot{D}}_{\rm{thre}}/{\dot{D}}_{i@12\rm{km},j}\right)$ and ${F}_{\rm{PEI}}\left({s}_{\rm{PEI},j}{\dot{D}}_{\rm{thre}}/{\dot{D}}_{i@12\rm{km},j}\right)$ for ${\dot{D}}_{\rm{thre}}$ = 80 μSv, are given in Table 4.

**Table 3:** Annual occurrence frequencies of the maximum SEP dose exceeding 1 mSv at the cruise altitudes of 12 km and 9 km for eight selected flight routes.

12 km	LAX_LHR	SYD_EZE	SFO_LHR	NRT_LHR	SYD_GIG	SYD_LIM	SYD_CPT	JFK_NRT
GLE60	0.0155	0.0111	0.0156	0.0123	0.0136	0.0047	0.0113	0.0117
GLE69	0.0128	0.0112	0.0118	0.0131	0.0134	0.0062	0.0134	0.0130
GLE70	0.0139	0.0193	0.0142	0.0183	0.0200	0.0085	0.0214	0.0189
GLE71	0.0149	0.0202	0.0167	0.0182	0.0198	0.0097	0.0225	0.0201
GLE72	0.0327	0.0299	0.0353	0.0323	0.0371	0.0082	0.0369	0.0361
Mean	0.0180	0.0183	0.0187	0.0188	0.0208	0.0075	0.0211	0.0200
Std	0.0083	0.0078	0.0094	0.0080	0.0097	0.0020	0.0101	0.0097

9 km	LAX_LHR	SYD_EZE	SFO_LHR	NRT_LHR	SYD_GIG	SYD_LIM	SYD_CPT	JFK_NRT
GLE60	0.0078	0.0056	0.0078	0.0062	0.0068	0.0024	0.0057	0.0060
GLE69	0.0062	0.0055	0.0058	0.0065	0.0066	0.0031	0.0066	0.0064
GLE70	0.0068	0.0098	0.0069	0.0093	0.0099	0.0047	0.0107	0.0093
GLE71	0.0072	0.0099	0.0080	0.0090	0.0098	0.0052	0.0110	0.0099
GLE72	0.0150	0.0137	0.0160	0.0140	0.0165	0.0041	0.0161	0.0159
Mean	0.0086	0.0089	0.0089	0.0090	0.0099	0.0039	0.0100	0.0095
Std	0.0036	0.0034	0.0041	0.0031	0.0040	0.0011	0.0041	0.0040

The mean values and the standard deviation (Std) of the frequencies obtained from the five selected GLE events are also summarized.

**Table 4:** Annual occurrence frequencies of the maximum SEP dose rate exceeding 80 μSv/h at the cruise altitudes of 12 km and 9 km for eight selected flight routes.

12 km	LAX_LHR	SYD_EZE	SFO_LHR	NRT_LHR	SYD_GIG	SYD_LIM	SYD_CPT	JFK_NRT
GLE60	0.0408	0.0293	0.0408	0.0384	0.0374	0.0165	0.0322	0.0344
GLE69	0.0440	0.0493	0.0440	0.0531	0.0493	0.0201	0.0335	0.0367
GLE70	0.0403	0.0800	0.0377	0.0641	0.0800	0.0439	0.0820	0.0703
GLE71	0.0521	0.0938	0.0775	0.0846	0.0938	0.0550	0.0938	0.0909
GLE72	0.0289	0.0263	0.0297	0.0241	0.0271	0.0146	0.0240	0.0299
Mean	0.0412	0.0557	0.0460	0.0529	0.0575	0.0300	0.0531	0.0524
Std	0.0083	0.0302	0.0184	0.0233	0.0284	0.0182	0.0323	0.0268

9 km	LAX_LHR	SYD_EZE	SFO_LHR	NRT_LHR	SYD_GIG	SYD_LIM	SYD_CPT	JFK_NRT
GLE60	0.0230	0.0162	0.0230	0.0215	0.0207	0.0092	0.0179	0.0196
GLE69	0.0240	0.0274	0.0240	0.0291	0.0274	0.0110	0.0190	0.0200
GLE70	0.0229	0.0469	0.0221	0.0385	0.0469	0.0274	0.0480	0.0405
GLE71	0.0304	0.0521	0.0429	0.0473	0.0521	0.0325	0.0521	0.0506
GLE72	0.0156	0.0138	0.0158	0.0109	0.0142	0.0080	0.0113	0.0158
Mean	0.0232	0.0313	0.0256	0.0295	0.0323	0.0176	0.0296	0.0293
Std	0.0053	0.0175	0.0102	0.0142	0.0165	0.0114	0.0189	0.0153

The mean values and the standard deviation of the frequencies obtained from the five selected GLE events are also summarized.

The tables showed that the frequencies deduced from the analysis of GLE69 are generally smaller than the others because SPE with hard spectra such as GLE69 tend to give lower aviation radiation doses compared to those with soft spectra at the same EII. However, the standard deviations of the frequencies are less than half of the corresponding mean values, indicating that the GLE dependences of the calculated frequencies are not too significant. This tendency suggests that the maximum SEP doses and dose rates for certain flight conditions can be roughly represented by EII and PEI, respectively, instead of the large uncertainties between the count rates of the polar neutron monitors and the spectral index of SEP as discussed in Asvestari et al.^14^. Among the eight selected flight routes, the largest mean frequencies are observed in the cases of SYD-CPT and SYD-GIG, which are 0.0211 and 0.0575 at 12 km for the dose and dose rate regulations, respectively. These results suggest that a GLE event that is strong enough to request a change in flight conditions occurs once per 47 and 17 years, respectively.

Evaluating all individual results shown in Tables 3 and 4, the frequency of exceeding the threshold value becomes higher when applied to the dose-rate criteria rather than the total dose criteria in most cases. Dose-rate regulation limits the maximum dose rate even for a short duration aviation route, which increases the sensitivity for risk, accordingly. Considering the fact that it is almost impractical to predict the total SEP dose during a GLE event, regulation based on dose-rate may give us a chance of avoiding significant exposure, as no aviation path reaches the threshold dose (1 mSv) among the five selected GLE cases and eight selected flight-routes.

Tables 5 and 6 show the annual risks calculated from Eqs. (3) and (4) multiplied with the worst-case-scenario scaling factor for daily-operated flight. The costs given in Table 1 and the frequencies shown in Tables 3 and 4 were used in the calculation. The mean values and the standard deviation of the risks obtained from the five selected GLE cases are also summarized in the tables. It is found from these tables that the mean annual risks estimated based on the dose and dose-rate regulations are less than 0.5 and 1.5 thousand USD, respectively, for all flight routes. These risks are not significantly large in comparison to the other aviation risks such as a volcanic eruption^29^. For example, many flights were cancelled when Eyjafjallajökull in Iceland erupted in 2010. During this eruption, the economic impact on aviation was estimated to be 1.7 billion USD^30^. Since the frequency of Icelandic volcanic eruptions was estimated to be 44 ± 7 years^31^, the annual risk of the Icelandic volcano eruption on aviation was calculated to be 38.6 million USD. This value is 10,000 times higher than the annual GLE risk for daily-operated long-distance flight obtained from this study.

**Table 5:** Annual risks in the units of 1000 USD/year to avoid the maximum SEP dose exceeding 1 mSv for eight selected flight routes calculated from Eq. (3) multiplied with the worst-case-scenario scaling factor for daily-operated flight.

	LAX_LHR	SYD_EZE	SFO_LHR	NRT_LHR	SYD_GIG	SYD_LIM	SYD_CPT	JFK_NRT
GLE60	0.37	0.25	0.36	0.29	0.29	0.11	0.25	0.27
GLE69	0.30	0.25	0.27	0.31	0.28	0.14	0.29	0.30
GLE70	0.33	0.44	0.32	0.44	0.42	0.20	0.47	0.43
GLE71	0.35	0.45	0.37	0.43	0.41	0.22	0.49	0.46
GLE72	0.74	0.63	0.75	0.69	0.73	0.18	0.74	0.75
Mean	0.42	0.40	0.41	0.43	0.43	0.17	0.45	0.44
Std	0.18	0.16	0.19	0.16	0.18	0.05	0.20	0.19

The mean values and the standard deviation of the frequencies obtained from the five selected GLE cases are also summarized.

**Table 6:** Annual risks in the units of 1000 USD/year to avoid the maximum SEP dose rate exceeding 80 μSv/h for eight selected flight routes calculated from Eq. (4) multiplied with the worst-case-scenario scaling factor for daily-operated flight.

	LAX_LHR	SYD_EZE	SFO_LHR	NRT_LHR	SYD_GIG	SYD_LIM	SYD_CPT	JFK_NRT
GLE60	1.08	0.75	1.07	1.04	0.91	0.42	0.81	0.91
GLE69	1.14	1.27	1.12	1.41	1.21	0.50	0.86	0.94
GLE70	1.07	2.13	1.02	1.82	2.01	1.19	2.14	1.88
GLE71	1.42	2.41	2.00	2.28	2.29	1.45	2.37	2.37
GLE72	0.74	0.65	0.74	0.56	0.64	0.37	0.55	0.75
Mean	1.09	1.44	1.19	1.42	1.41	0.79	1.35	1.37
Std	0.24	0.80	0.48	0.67	0.71	0.50	0.84	0.71

The mean values and the standard deviation of the frequencies obtained from the five selected GLE cases are also summarized.

## Conclusions

The risk assessment for the cost of countermeasures to reduce the radiation doses and dose rates due to SEP aviation exposure was performed in order to design an insurance product. In the assessment, the maximum SEP doses and dose rates for eight flight routes with two cruise altitudes during five GLE cases were evaluated by integrating the four-dimensional aviation dose rate data calculated by WASAVIES. Based on the results, the frequency that the total doses exceed 1 mSv or the dose rates exceed 80 μSv/h were estimated by scaling the magnitude of the GLE event using EII or PEI, respectively. Our calculations suggest that a GLE event of sufficient magnitude to request a change in flight conditions occurs once per 47 and 17 years in the case of following the dose and dose-rate regulations, respectively, and their conservatively-estimated annual risks associated with countermeasure costs are up to around 1.5 thousand USD for daily-operated long-distance flights. However, these results were derived from many simplifications such as constant flight speed and altitude during the cruise flight. Thus, more comprehensive risk assessments considering realistic flight schedules and detailed cost estimations must be conducted before an insurance system for aviation SEP exposure can be created.
